# Clearing cannabis criminal records: A survey of criminal record expungement availability and accessibility among US States and Washington DC that decriminalized or legalized cannabis

**DOI:** 10.1016/j.drugpo.2023.103983

**Published:** 2023-02-28

**Authors:** Tanner Wakefield, Stella Bialous, Dorie E. Apollonio

**Affiliations:** a Center for Tobacco Control Research and Education, School of Pharmacy, University of California, San Francisco, 530 Parnassus Avenue, Suite 366 Library, San Francisco, CA 94143, USA; b School of Nursing, University of California, San Francisco, 530 Parnassus Avenue, Suite 366 Library, San Francisco, CA 94143, USA; c Department of Clinical Pharmacy, University of California, San Francisco, 530 Parnassus Avenue, Suite 366 Library, San Francisco, CA 94143, USA

**Keywords:** Social justice, Criminal justice, Expungement, Cannabis, Public health

## Abstract

**Background:**

In 2022, despite expanding state-level legalization, cannabis remained illegal at the federal level, driving drug offenses, and contact with the justice system. Cannabis criminalization disproportionately impacts minorities, and criminal records carry negative economic, health, and social consequences. Legalization prevents future criminalization but fails to assist existing record-holders. We surveyed 39 states and Washington DC where cannabis was decriminalized or legalized to determine record expungement availability and accessibility for cannabis offenders.

**Methods:**

We performed a retrospective, qualitative survey of state expungement laws allowing record sealing or record destruction where cannabis use was decriminalized or legalized. Statutes were collected between February 25, 2021, and August 25, 2022, from state websites or NexisUni. We collected pardon information for two states from online state government resources. Materials were coded in Atlas.ti to determine if states had general, cannabis, and other drug conviction expungement regimes, petitions, or automated systems, waiting periods, and financial requirements. Codes were developed via inductive and iterative coding of materials.

**Results:**

Among places surveyed, 36 provided any conviction expungement, 34 provided general relief, 21 offered cannabis-specific relief, and 11 offered general drug relief, nonexclusively. Most states used petitions. Thirty-three general and 7 cannabis-specific programs required waiting periods. Nineteen general and 4 cannabis programs imposed administrative fees, and 16 general and one cannabis-specific program required legal financial obligations to be paid.

**Conclusion:**

Among the 39 states and Washington DC that decriminalized or legalized cannabis and offered expungement, more states relied on general rather than cannabis-specific systems, and the majority of these required record holders to petition for relief and imposed waiting periods and financial requirements. Research is needed to determine if automating expungement, reducing or eliminating waiting periods, and eliminating financial requirements may expand record relief for former cannabis offenders.

## Background

As of 2021, the United States criminalized and incarcerated more people than any other country (Highest to Lowest - Prison Population Total | World Prison Brief, n.d.; Prison Policy Initiative, 2021). One in three adults carries a criminal record,(Sibilla, 2020) and 105 million people have records in state crime databases (Selbin et al., 2017). Drug use and sales drive criminalization (Borden, 2016; Courtwright, 2004; Wildeman & Wang, 2017). In federal prisons, 45.3% of incarcerated people carry drug convictions (BOP Statistics: Inmate Offenses, n.d.). Cannabis, scheduled under the Controlled Substances Act as a Class I substance with a high abuse potential and no legitimate medical use (Drug Scheduling, n.d.), became the leading cause of drug arrests by 1996 (King & Mauer, 2006) and remains a major cause of arrest for possession offenses (Arrest Offense Counts in The United States, n.d.). Cannabis possession, distribution, or cultivation by the general public remains illegal at the federal level (Federal Laws and Penalties - NORML, 2022), despite increasing decriminalization, medical legalization, and recreational legalization at the state level since 2012.

Cannabis criminalization disproportionately harms minorities. African Americans composed 30% of cannabis arrests while comprising 14% of users between 1990 and 2002 (King & Mauer, 2006). Despite consuming cannabis at similar rates to Whites in 2018, African Americans were 3.64 times likelier to face arrest for cannabis offenses (Edwards et al., 2020). Criminal justice system contact negatively affects health (Binswanger et al., 2007; Massoglia & Remster, 2019), social welfare (Roberts, 2004; Wildeman & Wang, 2017), and economic outcomes (Pager, 2003). Holding a criminal record imposes over 44,000 potential consequences (Sewell & Paukstis, 2019). Criminal justice advocates have increasingly advocated for policies decriminalizing (Decriminalization - NORML, 2022) or legalizing (Legalization - NORML, 2022; Medical Marijuana Laws - NORML, 2022) cannabis possession and sales at the state level to reduce arrests; such policies prevent future harm but fail to assist existing record-holders. As a result, some states have provided general and cannabis-specific criminal record relief for former cannabis offenders (Berman, 2018).

Forty-five states and Washington DC allow some degree of expungement (50-State Comparison, 2020), which is defined as the destruction or sealing of criminal records (No Author, 2018). Expungement lowers recidivism (Prescott & Starr, 2020), enhances earnings and employability (Adams et al., 2017; Prescott & Starr, 2020), and improves people’s ability to obtain work, housing, and education funding (Beckett & Harris, 2011; Ispa-Landa & Loeffler, 2016; Schneider, 2018). Expungements are less expensive than work training programs (Prescott & Starr, 2020), and improve economic outcomes since holding a criminal record lowers employability and earnings over time (Beckett & Harris, 2011).

Expungement is relatively underused. A 2020 study found that under 20% of eligible people with a record of conviction in 10 states petitioned for expungement (Chien, 2020). This low uptake is driven by a lack of awareness, the complexity involved in navigating the expungement process, and regulatory and financial hurdles (Chien, 2020). In 2016, the American Bar Association found that 13 states required either payment of a fee for expungement, or payment of all existing fines or fees related to their conviction, to qualify for relief (Llorente, 2016). A majority of states’ expungement systems rely on petitions, although some, such as California, have automated expungement. Additionally, some states require that persons first obtain a Certificate of Eligibility to apply for expungement, which often requires its own process and fees. While some states offer expungement programs that address a broad range of offenses, other states offer expungement programs specific to cannabis records. There is limited research assessing how such programs operate in practice (Berman, 2018) or the extent to which record-clearing processes are automated across states (Chien, 2020).

We analyzed expungement statutes in states that decriminalized or legalized cannabis for medical and or recreational use to determine the availability and accessibility of expungement relief. We defined expungement as record sealing or destruction available to former cannabis offenders. We assessed whether states had automated or petition-based processes, as well as waiting periods, using model guidelines created by the nonprofit organization Alliance for Safety and Justice (Anderson et al., 2019). We also reviewed policies to identify whether states that provide conviction record relief offered general expungement, general drug expungement, or cannabis-specific expungement programs. We assessed whether states required Certificates of Eligibility to begin the expungement process, relied on pardons to grant relief, or vacated court rulings to provide expungement (meaning that the original verdict was eliminated or voided), a system known as vacatur. A review was also conducted of potential financial barriers, including administrative fees, and payments of existing financial obligations associated with convictions. Given the low expungement relief rates identified in previous research, we anticipated that most states would have expungement programs that allowed cannabis record relief, but that they would be petition-based and involve waiting periods and financial barriers.

## Methods

We conducted a retrospective qualitative survey of expungement laws in the US of states, and Washington DC, that had decriminalized or legalized cannabis use. Our goals were to determine (a) whether states allowed expungement of prior cannabis offenses, (b) whether states had generalized offense expungement regimes, general drug offense expungement regimes, cannabis-specific expungement regimes, or a combination of regimes, (c) whether they had automated or petition-based expungement, (d) the length of waiting periods (if any) required before seeking expungement relief, and (e) the existence of financial requirements for persons seeking relief. Our focus on expungement automation and waiting periods were informed by guidelines produced by the Alliance for Progress and Safety (Anderson et al., 2019).

As of September 2022, 26 states and Washington DC (hereafter included in the states count) had decriminalized cannabis possession (Decriminalization - NORML, 2022), 38 had legalized medical cannabis (Medical Marijuana Laws - NORML, 2022; State Medical Cannabis Laws, 2022) and 20 had legalized recreational cannabis (Hansen et al., 2022; Hartman, 2021; Legalization - NORML, 2022). We refer to the 39 states and Washington DC with some form of cannabis decriminalization or legalization, representing 40 jurisdictions, collectively referred to as “states” in this paper. To identify expungement policies, we collected each government’s statutes pertaining to general expungement, general drug expungement, and cannabis-specific expungement from state legislative websites or the NexisUni database. We also coded the pardon application for North Dakota to capture its provisions, a web page from the Pennsylvania Board of Pardons to obtain relevant data for cannabis-specific pardon relief, and legislative text applying to cannabis expungement provisions in Vermont when the statute itself could not be located. Search terms included “cannabis”, “marijuana”, “expungement”, “record seal”, “set aside”, “vacatur”, legalization”, “recreational marijuana”, “retail marijuana”, “medical marijuana”, “decriminalization”, “statute”, and “pardon.” Google searches using these terms in combination with each state were used to find statutes from state government websites. Statutes that were not found through a web search were triangulated using government or legal websites that provided statute codes or names, then accessed through state legislative websites or NexisUni. If a state had expiring statutes that would be superseded by a new statute, we excluded the expiring statute and analyzed the new legislation. Only Washington State was in this category (its statute took effect during the period of data collection).

Statutes were selected if they were relevant to general, drug, or cannabis-specific expungement regimes related to convictions, wait periods, fees, and fines. The research was approved by the University of California, San Francisco Institutional Review Board (#10–01262). The research used data that can be accessed freely by the public without special permission or application, the information was defined as not “private” and not involving human subjects. One author (TW), who had previously written a report on state tobacco control policymaking that required interpreting legal regulations, and analyzing their impacts (Wakefield & Glantz, 2020), imported the text of each statute into Atlas.ti for descriptive coding between February 25, 2021, and August 25, 2022. Statutes were iteratively coded to determine the characteristics and restrictions of state expungement programs until default codes were developed for program component themes. The identified themes included the type of substance (cannabis, other), the mechanism (petition, expungement), waiting period (presence, duration), and financial requirements (presence, type—e.g., filing, administrative). We indicated whether expungement was automated, and the presence and length of waiting periods for all states, using guidelines created by the Alliance for Progress and Safety (Anderson et al., 2019). Details relevant to waiting periods were further refined through a review of records. The resulting codes categorized statutes by whether they targeted relief for convictions, general offenses, general drug offenses, cannabis-specific offenses, or provided non-conviction expungement mechanisms. We classified programs providing relief for cases that were deferred in exchange for probation, or for participation in treatment programs, as “conviction expungement” since a sentence was assigned, and served, to avoid entry of a guilty verdict into government records. Waiting periods were categorized by expungement program type, by their duration, and by the level of offense targeted (e.g., violations, misdemeanors, and felonies). Financial requirements were coded to reflect whether they constituted an administrative fee or involved a financial obligation related to conviction(s) that had to be resolved before expungement relief was received. The research team reviewed a subset of initial statutes together, then after an agreement was reached regarding themes and categorizations, the remaining statutes were read and reviewed by a single author (TW). When there was uncertainty regarding the coding or categorization of a statute or law, the authors discussed it until they reached a consensus.

## Results

a States allowing expungement of prior cannabis offenses

### Expungement

We found that 36 of the 40 states with some level of cannabis decriminalization or legalization in 2022 offered some type of conviction expungement relief. Four states that had decriminalized or legalized cannabis use (Alaska, Connecticut, Florida, and Maine) did not offer conviction record expungement, as shown in Table 1. Among the 36 states that had expungement programs permitting clearance of cannabis conviction records, 34 offered general expungement programs, 21 had created cannabis-specific programs, and 11 of 36 offered general drug offense expungement. Multiple states offered a combination of expungement processes based on offense type (i.e., general expungement processes and cannabis offense processes, or general processes and drug offenses processes, or all three).

a States with generalized offense expungement regimes, general drug offense expungement regimes, cannabis-specific offense expungement regimes, or a mixture of regimes, and whether they had automated or petition-based expungement

### General and general drug expungement programs

Of the 34 states offering general expungement, 33 provided petition-based mechanisms and 9 offered automated mechanisms. Of the 9 with automated mechanisms (including via court motion), 8 also permitted record holders to submit petitions. Seven states nonexclusively enabled or provided expungement via pardons.

Eleven states offered general drug offense expungement; of those targeting general drug expungement, 9 provided petition-based expungement and 3 states offered automated expungement through the courts or prosecutorial motions (one used both). Two of 3 states that offered automated expungement of general drug offenses did not accept petitions. Results are provided in Table 2.

### Cannabis-specific expungement programs

The 21 states with cannabis-specific expungement programs were more likely to provide both automated and petition-based mechanisms, relative to states with general expungement programs. Of those 21, 17 provided petition-based mechanisms, 9 offered automated mechanisms, and 5 used pardons for expungement (these categories are nonexclusive as some states utilize multiple mechanisms and or programs, and some automated mechanisms rely partially or completely on pardons). Seven states provided multiple methods to expunge cannabis records (i.e., petition plus automated, petition plus pardon). Among the 21 states with cannabis expungement programs, 13 had legalized recreational use, while 8 permitted medical use (six of those medical states also decriminalized possession).

a Waiting periods for people seeking expungement

### Waiting periods

Thirty-three of the 36 states offering expungement established waiting periods for clearing general criminal records. All 33 had general offense waiting periods, while seven states imposed waiting periods for cannabis-specific offenses, as shown in Table 3.

### Waiting periods for general expungement

General expungement waiting periods could extend up to 20 years. For violations and infractions, waiting periods ranged from less than 1 year to 5 years, for misdemeanors waiting periods ranged from less than 1 year to 10 years, and for felonies waiting periods ranged from 1 year to 20 years (Table 3). States often set waiting periods that varied by offense and severity (i.e., violation, misdemeanor or felony combined with violent, nonviolent, or specific crime types). In total, 33 governments set waiting periods for petition expungement, generally setting multiple durations based on offense level and type. The most common waiting periods were 1–2 years (10 states), 2–5 years (28 states), and 10 or more years (17 states).

Five states set waiting periods before people received automated expungement. Michigan automatically expunged general misdemeanor convictions after 7 years and felonies after 10 years, Pennsylvania expunged misdemeanors after 10 years, South Dakota expunged violations and misdemeanors at 5 years, and Vermont expunged convictions within 30 days for people who were between the ages of 18–21 years at the time they were charged. Arkansas permitted courts to immediately expunge an offender’s record after completing drug or other court-ordered treatment (which could also be requested by petition).

Four states explicitly established waiting periods (or lack thereof) for expungements after a pardon. Two states, Colorado and Illinois, allowed expungement at any time following a pardon. Alabama allowed expungement 180 days after a felony pardon, and Maryland required a 10-year waiting period before records associated with pardoned offenses were expunged.

### Waiting periods for cannabis expungement

The 7 states that set waiting periods for cannabis-specific expungement programs set them to shorter durations relative to waiting periods for general expungement, as shown in Table 4. The 6 states with petition mechanisms set waiting periods ranging between no wait to 4 years. The 3 states with automated expungement waiting periods set waiting periods ranging between 1–2 years or tied waiting periods to the date of the offense. Three states with automated expungement, California, Illinois and New Mexico, set waiting periods for the expungement of cannabis records. Illinois automatically expunged certain cannabis-related offenses after 1 year, while California and New Mexico expunged certain cannabis-related offenses after 2 years. Illinois expunged cannabis offenses dated between 2013 and 2019 by 2021, offenses dated between 2000 and 2013 by 2023, and offenses dated before 2000 by 2025. Arizona and New Jersey imposed cannabis-specific waiting periods for expungement, with New Jersey also requiring a 3-year wait, completion of probation, or resolution of financial assessments, for cannabis offenses involving distribution or intent to distribute.

a Financial requirements for persons seeking relief

### Financial costs

Of the 34 states offering general expungement programs, 19 required that people pay administrative fees to procure relief, as shown in Table 5. Administrative fees collectively refer to filing and processing fees incurred during the expungement process. Among these 19, 11 charged a filing fee, 13 instituted a processing fee, and 5 states required both a filing fee and a processing fee. Nine of these 19 states charging administrative fees offered waivers for indigence. Oklahoma reimbursed filing fees upon expungement, and West Virginia waived administrative fees if an applicant participated in a treatment or diversion program. Nevada and Rhode Island did not charge administrative fees for expunging offenses that had been decriminalized at the state level.

Compared to general expungement programs, cannabis-specific expungement programs were less likely to require payment of administrative fees. Among the 21 states with cannabis-specific expungement programs, only 4 required payment of a filing or processing fee (Table 5). Three states required payment of filing fees to have records expunged, and 2 others required payment of processing fees. Delaware was the only state that set both filing and processing fees. Arizona offered an indigent waiver for cannabis offenses, and Virginia reimbursed filing fees after expungement. Three states required that a person seeking expungement first obtain a Certificate of Eligibility: Illinois, Louisiana, and Utah. Of these three, Utah imposed associated administrative fees but waived certificate requirements for offenses involving cannabis possession as well as for persons previously charged for using cannabis for qualifying health conditions. None of the pardon-based expungement programs required that pardon petitioners or recipients pay fees to receive an expungement.

Seventeen states required that people seeking expungement pay other legal financial obligations to secure relief, as shown in Table 6. Of these, 16 required that any outstanding financial judgments, legal obligations, and restitution be paid before granting general expungement, and New Jersey required these obligations be paid before granting cannabis-specific relief (Table 6). Although Oregon historically required that applicants for cannabis record expungement pay outstanding financial obligations, the state repealed that requirement in 2022.

Only Illinois explicitly permitted the expungement of records if financial obligations remained unpaid. Delaware and Rhode Island permitted courts to waive requirements to pay existing legal obligations. Colorado vacated (eliminated) underlying convictions as part of its expungement process, and voided remaining fees, fines, and restitution associated with vacated cases.

## Discussion

Among the 39 states and Washington DC that decriminalized or legalized cannabis use, we found that 36 permitted some type of expungement of conviction records related to cannabis and four states prohibited the expungement of existing convictions. Within the 36 states that allowed expungement, 34 offered general expungement, 21 offered cannabis-specific expungement, and 11 offered general drug offense expungement (some states offered more than one program). Among the 34 states with general expungement programs, 33 allowed expungements by petition, 9 expunged records automatically (8 states offered both petition-based and automatic expungement), and 7 allowed pardons as part of records expungement. Among the 21 states with cannabis-specific expungement programs, 17 allowed expungements by petition, 9 expunged records automatically, 5 allowed pardons, and 7 provided multiple expungement programs.

States with petition systems require applicants to navigate complex procedures that consume time and resources (Murray, 2021; Prescott & Starr, 2020). Administrative burdens are broadly defined as the compliance, learning, and psychological costs endured by persons interacting with civic institutions (Moynihan et al., 2015) that are correlated with reductions in uptake of government services (Ray et al., 2022). Petition systems increase administrative burdens, and as a result navigating them may require record holders to secure legal assistance, further increasing costs and limiting relief for people who are socioeconomically disadvantaged due to their convictions (Heinrich, 2016; Murray, 2021). Cannabis laws have imposed disproportionate criminal burdens on minorities, making accessible expungement important to improving racial equity outcomes as cannabis legalization expands (Crawford, 2021). Limited knowledge of expungement, the education needed to navigate petition systems, stress incurred during the process, the requirement to wait a specific period before seeking relief, and the payment of fees likely contributes to limited use of petition-based expungement (Berman, 2018; Moynihan et al., 2015). State policymakers may create, or retain, systems based on petitions that make expungement more difficult in the belief that this will encourage lawful behavior, and that it will prevent those who are likely to reoffend from having their records expunged (Chien, 2020). However, research suggests that expungement reduces recidivism (Prescott & Starr, 2020), improves earnings, and increases employability (Adams et al., 2017; Prescott & Starr, 2020) by removing criminal records that limit the ability to obtain work, housing, or secure education funding (Beckett & Harris, 2011; Ispa-Landa & Loeffler, 2016; Schneider, 2018). Expungements are also low-cost relative to job training programs (Prescott & Starr, 2020) and improve economic outcomes, as persons with criminal records earn less over time (Beckett & Harris, 2011). Our findings suggest that potential beneficiaries may have been denied this economic and social relief, since the majority of states require record holders to petition for expungement.

The majority of expungement programs also imposed administrative burdens in the form of waiting periods and fees. Administrative burdens can compound racial disparities associated with stigma, and even small administrative burdens can limit access to government aid and services (Ray et al., 2022). For example, waiting periods established by some states, in the expectation that these will ensure only “truly reformed” persons obtain record relief, can perpetuate inequalities as racial minorities are disproportionately criminalized by cannabis offenses (Ray et al., 2022).

Among the states offering expungement, all but two had waiting periods, which varied in duration by offense levels and types. Waiting periods are administrative burdens because they create a psychological hurdle and sustain stress related to criminal record stigma and penalties (Prescott & Starr, 2020). Shorter waiting periods improve record-holders’ long-term earnings (Selbin et al., 2017), while longer waiting periods can increase recidivism by hindering reentry into employment. Some states have reduced waiting periods to improve access to expungement (Murray, 2021). Waiting periods could arguably be eliminated for former cannabis offenders in states where use is legal and or decriminalized, given that criminalization has already been overturned (Berman, 2018).

We also found that most states required applicants to pay administrative fees and resolve outstanding judgments and legal financial obligations, pay restitution, or all of the above. Fees constitute another burden, either directly by increasing financial costs or indirectly by requiring applicants to expend time to learn how to navigate indigency waiver processes. We were unable to find prior research assessing the impacts of administrative fees on cannabis-related record holders or applicants for expungement. However, legal financial penalties disproportionately burden minorities and persons of lower socioeconomic status (Bing et al., 2022) and are associated with increased poverty (O’Neill et al., 2022). Although multiple states permit courts to grant indigency waivers, judges do not always allow this assistance (Slavinski & Spencer-Suarez, 2021). Many record holders are unaware of indigency waivers, creating an invisible barrier to expungement (Slavinski & Spencer-Suarez, 2021). The inability to pay financial penalties may also inflict collateral consequences, such as driver’s license revocation or having debt reported to credit agencies (Martin et al., 2018). States could reduce these harms by eliminating administrative fees for expungement and postponing or eliminating payment of outstanding legal financial obligations.

Previous research has suggested that states explore automated expungement systems to reduce administrative burdens (Chien, 2020; Starr, 2020) and increase rates of expungement (Moynihan et al., 2015; Prescott & Starr, 2020; Starr, 2020). Reducing administrative burdens has historically increased records expungement (Heinrich, 2016). Nonetheless, further research is needed on automatic expungement programs. For example, California’s historic petition-based expungement program for cannabis offenses resulted in 5% to 7% of eligible candidates applying (Chien, 2020). The state implemented automated expungement for cannabis offenses in 2018 (Komp, 2020) to provide greater relief (Schnell, 2018), but progress on automatically clearing the 220,000 cannabis conviction records estimated to qualify for clearance has been uneven (Marijuana Moment, 2021). As of July 2021, 34,000 records remained unprocessed, spurring the introduction of new legislation to provide further automatic relief by January 1, 2023 (Feldman, 2022).

Our study has limitations. It focused on expungement for cannabis offenses in states that decriminalized or legalized cannabis use and sales by September 2022 and cannot be generalized to other offenses. People with cannabis offenses are more likely to secure records expungement than people with non-cannabis offenses, given the growing social permissiveness around cannabis and the expansion of legalization. States also have expungement practices that this study did not capture, such as filing and dissemination requirements for expungement petitions and orders, mandatory hearings, imposing a burden of proof on applicants, or program qualifiers limiting expungement to either first-time offenders or persons that are diverted to and complete probation or drug treatment. In addition, we did not explore regulations that prevent legal aid organizations from filing petitions or other factors that could increase or reduce expungement access, such as prohibiting relief for people with unrelated offenses, imposing caps on total offenses, or on certain types of offenses. These requirements are administrative burdens that further complicate the process of records expungement.

Although we cataloged expungement provisions pertaining to violations, misdemeanors, and felonies, we did not survey the levels and types of cannabis offenses in each state or how those may have changed over time (i.e., a cannabis offense may have first been a felony, then a violation after decriminalization, then not a crime at all after legalization). Instead, our paper surveyed all potentially applicable statutes and restrictions that could apply to a cannabis offense. Finally, cannabis is a dynamic policy area and legalization laws as well as expungement statutes have changed rapidly since 2012. Nonetheless, this research suggests how current expungement programs may affect the ability of cannabis-related record holders to secure relief in states that have attempted to reduce cannabis criminalization, and provides the ground-work for further exploration.

## Conclusions

Nationally, in 2016, 35% of eligible non-convicted persons in the US still carried clearable records (Chien, 2020). Holding a record is a social and economic burden (Ispa-Landa & Loeffler, 2016; Pager, 2003), and expunging records improves employment and social outcomes (Adams et al., 2017; Prescott & Starr, 2020; Selbin et al., 2017). The social harms of cannabis prohibition have led to efforts to decriminalize and legalize cannabis, but although legalization prevents future criminal charges, it does not assist persons charged under prior policies. Although the majority of the 39 states and Washington DC that had legalized or decriminalized cannabis by 2022 had an expungement process that could be applied to cannabis offenses, these programs typically applied to all types of offenses, were petition-based and involved waiting periods and payment of administrative fees. Further research should examine whether states can increase rates of expungement by reducing administrative burdens. Potentially promising strategies include automated expungement, reducing or eliminating waiting periods, and eliminating requirements to pay fees and fines.

## Figures and Tables

**Table 1:** Expungement programs in states that had decriminalized or legalized cannabis in some form.

State	Recreational (*Legalization - NORML,* 2022)	Medical (*Medical Marijuana Laws - NORML,* 2022)	Decriminalization (*Decriminalization - NORML,* 2022)	General expungement program	Cannabis expungement program	General drug offense expungement

Alabama	No	Yes	No	Yes	No	Yes
Alaska	Yes	Yes	Yes	No	No	No
Arizona	Yes	Yes	No	Yes	Yes	No
Arkansas	No	Yes	No	Yes	No	Yes
California	Yes	Yes	Yes	Yes	Yes	No
Colorado	Yes	Yes	Yes	Yes	Yes	Yes
Connecticut	Yes	Yes	Yes	No	No	No
Delaware	No	Yes	Yes	Yes	Yes	No
Florida	No	Yes	No	No	No	No
Hawaii	No	Yes	Yes	Yes	Yes	Yes
Illinois	Yes	Yes	Yes	Yes	Yes	Yes
Louisiana	No	Yes	Yes	Yes	No	No
Maine	Yes	Yes	Yes	No	No	No
Maryland	No	Yes	Yes	Yes	Yes	Yes
Massachusetts	Yes	Yes	Yes	Yes	No	No
Michigan	Yes	Yes	No	Yes	Yes	No
Minnesota	No	Yes	Yes	Yes	Yes	Yes
Mississippi	No	Yes	Yes	Yes	No	No
Missouri	No	Yes	Yes	Yes	No	No
Montana	Yes	Yes	No	Yes	Yes	No
Nebraska	No	No	Yes	Yes	No	Yes
Nevada	Yes	Yes	Yes	Yes	No	No
New Hampshire	No	Yes	Yes	Yes	Yes	No
New Jersey	Yes	Yes	No	Yes	Yes	No
New Mexico	Yes	Yes	Yes	Yes	Yes	No
New York	Yes	Yes	Yes	Yes	Yes	No
North Carolina	No	No	Yes	Yes	No	Yes
North Dakota	No	Yes	Yes	No	Yes	Yes
Ohio	No	Yes	Yes	Yes	No	No
Oklahoma	No	Yes	No	Yes	No	No
Oregon	Yes	Yes	Yes	Yes	Yes	No
Pennsylvania	No	Yes	No	Yes	Yes	No
Rhode Island	Yes	Yes	Yes	Yes	No	No
South Dakota	No	Yes	No	Yes	No	No
Utah	No	Yes	No	Yes	Yes	No
Vermont	Yes	Yes	Yes	Yes	Yes	Yes
Virginia	Yes	Yes	Yes	No	Yes	No
Washington, DC	Yes	Yes	Yes	Yes	No	No
Washington State	Yes	Yes	No	Yes	Yes	No
West Virginia	No	Yes	No	Yes	No	No
Totals	20	38	27	34	21	11

**Table 2:** Cannabis expungement program mechanisms used by each state.

State	Petition	Auto	Pardon (provides or enables expungement)	Any

Alabama	No	No	No	No
Alaska	No	No	No	No
Arizona	Yes	No	No	Yes
Arkansas	No	No	No	No
California	Yes	Yes*	Yes*	Yes
Colorado	No	No	Yes	Yes
Connecticut	No	No	No	No
Delaware	Yes	Yes	No	Yes
Florida	No	No	No	No
Hawaii	Yes	No	No	Yes
Illinois	Yes	Yes*	Yes*	Yes
Louisiana	No	No	No	No
Maine	No	No	No	No
Maryland	Yes	No	No	Yes
Massachusetts	No	No	No	No
Michigan	Yes	No	No	Yes
Minnesota	Yes	No	No	Yes
Mississippi	No	No	No	No
Missouri	No	No	No	No
Montana	Yes	No	No	Yes
Nebraska	No	No	No	No
Nevada	No	No	No	No
New Hampshire	Yes	No	No	Yes
New Mexico	No	Yes	No	Yes
New Jersey	Yes	Yes	No	Yes
New York	Yes	Yes	No	Yes
North Carolina	No	No	No	No
North Dakota	Yes	Yes	Yes	Yes
Ohio	No	No	No	No
Oklahoma	No	No	No	No
Oregon	Yes	No	No	Yes
Pennsylvania	No	No	Yes**	Yes
Rhode Island	No	No	No	No
South Dakota	No	No	No	No
Utah	Yes	No	No	Yes
Vermont	No	Yes	No	Yes
Virginia	Yes	Yes	No	Yes
Washington, DC	No	No	No	No
Washington State	Yes	No	No	Yes
West Virginia	No	No	No	No
Totals	17	9	5	21

* Automated cannabis conviction relief uses pardon as primary or secondary mechanism to provide relief.

** A pardon automatically expunges records held by the FBI or Pennsylvania State Police, although a petition is needed to ensure that criminal records are expunged from the web portal of the Unified Judicial System of Pennsylvania. (Frequently Asked Questions, n.d.)

**Table 3:** Wait period frequency for general petition and automatic expungement programs.

Petition	Conviction-based wait periods	Violations	No wait	<1 year	1–2 years	2–5 years	6–9 years	10+ years	Total
			0	1	4	7	0	0	12

		Misdemeanors	1	2	7	29	4	3	46
		Felonies	0	0	1	20	7	13	41
		No subsequent misdemeanor or felony within certain time	0	0	0	1	1	0	2
		Unspecified offense level	2	0	0	11	0	0	2
		Overturned convictions	1	1	3	0	0	7	24
		Unspecified offense level (crime committed while underage)	1	0	0	2	0	0	3
	Conviction deferral, dismissal and diversion	Deferral (any)	1	0	3	1	0	0	5
		Deferral with diversion or treatment	3	0	1	1	0	0	5
Auto	Conviction-based wait periods	Violations	0	0	0	1	0	1	2
		Misdemeanors	0	0	0	1	1	0	2
		Felonies	0	0	0	0	0	1	1
		Unspecified offense level	1	1	0	0	0	0	1
Pardons		Felonies	0	1	0	0	0	0	1
		Unspecified	1	0	0	0	0	1	2
Expungement reapplication	Conviction-based wait periods	Violations	0	1	0	0	0	0	1
		Misdemeanors	0	1	0	0	0	0	1
		Felonies	0	0	0	0	0	0	0
		Unspecified	0	0	1	5	0	0	6
Total	11	7	20	80	13	23			

* Figures include drug offense wait periods and a wait period to apply for a certificate of eligibility to seek expungement.

** Laws may be counted as both automatic and petition based if it allows court to grant without petition while allowing an individual to also petition.

*** Figures were tallied based on general offense level and year. For instance, two misdemeanor-associated wait periods of 2 years were counted as one. Two misdemeanor-level wait periods with different wait lengths set by a single state counted as two (e.g., one 3-year misdemeanor wait and one 4-year misdemeanor wait in a state counts as 2 in the 2–5 year bracket).

**Table 4:** Wait period frequency for cannabis petition and automatic expungement programs.

Mechanism	Conviction or Diversion Type	Offense or diversion level	No wait	1 year	2 years	3 years	4 years	Offenses cleared automatically by certain year

Petition	Conviction-based wait periods	Unspecified offense level	2	1	1	1	1	0
	Conviction diversion-based wait periods	Deferred with probation or supervision (No entry of guilt entered into record in exchange for completing probation or court ordered supervision)	0	1	0	0	0	0
Automated	Conviction-based wait periods	Unspecified offense level	0	0	2	0	0	Offenses committed between
2013–2019 expunged by 2021								
2000–2013 expunged by 2023								
Before 2000 expunged by 2025								
	Conviction diversion-based wait periods	Deferred with probation or supervision (No entry of guilt entered into record in exchange for completing probation or court ordered supervision)	0	1*	0	0	0	0

* Applies to dismissed or vacated minor cannabis offenses.

**Table 5:** Frequency of stated fee requirements.

		General	Cannabis	Certificate of Eligibility	Pardons

Convictions	Any fee	19	4	2	0
	Any filing fee (administrative)	11	3	1	0
	Any processing fee (administrative)	13	2	1	0
	Any fee waiver	10	1	0	0
	Fees waivable	1	0	0	0
	Indigent Fee Waiver	9	1	0	0
	Filing fee reimbursed upon success	1	1	0	0
	Filing fee waived for treatment or diversion participation	1	0	0	0
	No offense reduction fee level	0	1	0	0
	Statement declares no fees	1	1	0	2
	Statement declares no filing fee for decriminalized offenses	2	0	0	0
	No fees for dismissal of active sentence	0	1	0	0
Deferred or dismissed cases	Filing Fee for deferred cases	1	0	0	0
	Processing fee for deferred cases	1	0	0	0
	Dismissed case fees waivable	1	0	0	0
	Indigent fee waiver for dismissed cases	1	0	0	0

**Table 6:** States restricting expungement based on existence of unpaid financial judgments, obligations or restitution of statutes.

State	States restricting expungement for outstanding financial requirements			States with expungement exceptions for outstanding financial requirements		States with provisions to vacate outstanding financial requirements		
	States with restrictions	General program	Cannabis program	Outstanding legal obligations do not prohibit expungement	Financial legal obligation requirements may be waived	Vacatur of remaining financial obligations	Vacatur of case voids associated fees, fines or restitution	Vacatur of remaining financial obligations for active cannabis sentences following legalization

Alabama	Yes	Yes	No	No	No	No	No	No
Arizona	Yes	Yes	No	No	No	No	No	No
Arkansas	Yes	Yes	No	No	No	No	No	No
Colorado	Yes	Yes	No	No	No	Yes	Yes	No
Delaware	Yes	Yes	No	No	Yes	No	No	No
Illinois	Yes	Yes	No	Yes	No	No	No	No
Missouri	Yes	Yes	No	No	No	No	No	No
Montana	Yes	Yes	No	No	No	No	No	No
New Jersey	Yes	No	Yes	Yes	No	Yes	No	Yes
New Mexico	Yes	Yes	No	No	No	No	No	No
North Carolina	Yes	Yes	No	No	No	No	No	No
Oklahoma	Yes	Yes	No	No	No	No	No	No
Pennsylvania	Yes	Yes	No	No	No	No	No	No
Rhode Island	Yes	Yes	No	No	Yes	No	No	No
Utah	Yes	Yes	No	No	No	No	No	No
Vermont	Yes	Yes	No	No	No	No	No	No
Washington State	Yes	Yes	No	No	No	No	No	No
Totals	17	16	1	1	2	2	1	1
